# Ergothioneine-Mediated Neuroprotection of Human iPSC-Derived Dopaminergic Neurons

**DOI:** 10.3390/antiox13060693

**Published:** 2024-06-05

**Authors:** Damien Meng-Kiat Leow, Irwin Kee-Mun Cheah, Lucrecia Chen, Yang-Kai Ng, Crystal Jing-Jing Yeo, Barry Halliwell, Wei-Yi Ong

**Affiliations:** 1 Department of Anatomy, Yong Loo Lin School of Medicine, National University of Singapore, Singapore 117594, Singapore; 2 Neurobiology Research Programme, Life Sciences Institute, National University of Singapore, Singapore 117456, Singapore; 3 Department of Biochemistry, Yong Loo Lin School of Medicine, National University of Singapore, Singapore 117596, Singapore; 4 Institute of Molecular and Cell Biology (IMCB), Agency for Science, Technology and Research (A*STAR), Singapore 138673, Singapore; 5 National Neuroscience Institute (NNI), Singapore 308433, Singapore; 6 Institute of Education in Healthcare and Medical Sciences, School of Medicine, University of Aberdeen, Aberdeen AB51 7HA, UK; 7 Department of Neurology, Boston Children’s Hospital, Harvard Medical School, Boston, MA 02115, USA; 8 Department of Neurology, Feinberg School of Medicine, Northwestern University, Evanston, IL 60611, USA; 9 Lee Kong Chian School of Medicine, Nanyang Technological University, Singapore 308232, Singapore

**Keywords:** neurodegeneration, ergothioneine, mitochondrial dysfunction, 6-OHDA, Parkinson’s disease

## Abstract

Cell death involving oxidative stress and mitochondrial dysfunction is a major cause of dopaminergic neuronal loss in the substantia nigra (SN) of Parkinson’s disease patients. Ergothioneine (ET), a natural dietary compound, has been shown to have cytoprotective functions, but neuroprotective actions against PD have not been well established. 6-Hydroxydopamine (6-OHDA) is a widely used neurotoxin to simulate the degeneration of dopaminergic (DA) neurons in Parkinson’s disease. In this study, we investigated the protective effect of ET on 6-OHDA treated iPSC-derived dopaminergic neurons (iDAs) and further confirmed the protective effects in 6-OHDA-treated human neuroblastoma SH-SY5Y cells. In 6-OHDA-treated cells, decreased mitochondrial membrane potential (ΔΨm), increased mitochondrial reactive oxygen species (mROS), reduced cellular ATP levels, and increased total protein carbonylation levels were observed. 6-OHDA treatment also significantly decreased tyrosine hydroxylase levels. These effects were significantly decreased when ET was present. Verapamil hydrochloride (VHCL), a non-specific inhibitor of the ET transporter OCTN1 abrogated ET’s cytoprotective effects, indicative of an intracellular action. These results suggest that ET could be a potential therapeutic for Parkinson’s disease.

## 1. Introduction

Parkinson’s disease (PD) is one of the most prevalent neurological diseases globally (second after Alzheimer’s disease) [1], affecting approximately 2% to 3% of people aged 65 years and above [2]. There is increased oxidative damage in the affected brain region, involving iron–iron dependent generation of reactive oxygen species (ROS) [3,4,5,6,7,8]. Mitochondrial dysfunction is closely related to the pathophysiology of PD. Brain samples from PD patients showed evidence of mitochondrial damage [9,10,11] and mitochondrial complex I and II defects were detected in the substantia nigra pars compacta (SNc) of patients with PD [12,13,14,15]. Mitochondrial modulators with neuroprotective potential are currently being explored for treatment of PD. These include creatine (N-aminoiminomethyl-N-methylglycine) and coenzyme Q10 (CoQ10), which have shown some neuroprotective effects [16,17,18].

Ergothioneine (ET) is a naturally occurring amino thione synthesised by certain fungi and bacteria. It has gained much attention recently due to its cytoprotective, including neuroprotective, effects [19,20,21]. ET is an antioxidant that scavenges certain ROS [19,22,23] and chelates pro-oxidant divalent metal ions [21]. It can cross the blood–brain barrier (BBB) and accumulate in the brain following oral administration to mice [24], and is also found in the human brain [19,22,25]. ET has shown protective effects against oxidative damage by oxysterols in human brain endothelial cells [26,27], in rodent models of stroke [28], cardiovascular disease [29,30], inflammation [31,32], cognitive impairment and dementia [33,34,35], and aging [36]. ET levels in the blood are lower in PD patients [37]. Dietary ET is taken up in the gut before entering cells via the organic cation transporter novel type-1 (OCTN1) [38,39]. The latter can be inhibited by verapamil hydrochloride, VHCL, a non-specific ion channel inhibitor [26]. Knockout mice that lack OCTN1 are more susceptible to inflammation and oxidative damage [40].

Discovery of potential mitochondrial protectants that could cross the BBB could be highly valuable, given the role of mitochondrial dysfunction in PD [11,12,13,14,15,41,42]. Yuzawa et al. (2024) [43] reported the protective effect of ET against 6-OHDA neurotoxicity in immortalised mouse hypothalamic cells (GT-17) through reduction in endoplasmic reticulum stress response. Thus far, however, it is not known if ET could protect against damage using cell models more relevant to human PD, such as human induced pluripotent stem cell (hiPSC)-derived dopaminergic neurons. Dopaminergic cultures produced from hiPSCs have been demonstrated to replicate the development of the midbrain, and Parkinsonian cellular networks can be formed from neurons that survive and integrate [44]. 6-hydroxydopamine (6-OHDA) has been used to induce degeneration of dopaminergic neurons in cellular models of PD [45,46] and in vivo in rodents [47]. 6-OHDA enters dopaminergic neurons via dopamine transporters (DAT), leading to mitochondrial dysfunction, increased oxidative damage and cell death [48,49]. In this study, we sought to determine whether ET could protect against 6-OHDA-induced cell death and mitochondrial damage in tyrosine hydroxylase positive (TH+) cells in vitro. TH is a marker for dopamine-containing neurons [50]. Human iPSC-derived dopaminergic neurons and human-derived neuroblastoma SH-SY5Y cells were chosen as they are widely used as a cellular model for the investigation of neuronal differentiation and neuroprotection events, including PD [44,51,52,53].

## 2. Materials and Methods

### 2.1. Chemicals

Ergothioneine (ET) was provided by Tetrahedron (Paris, France). 6-Hydroxydopamine hydrobromide (6-OHDA) and Verapamil hydrochloride (VHCL) were purchased from Sigma Aldrich (St. Louis, MO, USA).

### 2.2. Cell Culture

To make a 2 mM stock solution, 1 mg of 6-OHDA was dissolved in 2 mL of 0.1% ascorbic acid solution in ice-cold MiliQ water. The solution was filtered and can be stored at −20 °C for up to 1 week. SH-SY5Y cells were cultured in a 5% CO_2_ humidified incubator at 37 °C in high-glucose Dulbecco’s modified Eagle medium (DMEM-H) with 10% fetal bovine serum (FBS) including glutamine and sodium pyruvate, and 1% penicillin-streptomycin. Cells were then cultured in either 6-well plates (qPCR/Immunofluorescence), 12-well plates (Flow cytometry/ATP assay), or 96-well plates (MTT assay) for 24 h and at 80–90% confluency prior to treatments.

### 2.3. Pharmacological Patterning of Dopaminergic Neurons

The embryonic stem cells (ESCs) and healthy-patient-derived induced pluripotent stem cells used in this study are BJ, H9, and GM23720 from Corning (New York, NY, USA). The induction of iDAs from stem cells was performed as described in [54]. In brief, stem cells were cultured on Matrigel-coated plates in StemMACS iPS-Brew XF (130104368) (Miltenyi Biotec, Bergisch Gladbach, Germany). On the day of differentiation (D0), stem cells were treated with Accutase (12679-54) (Nacalai Tesque, Kyoto, Japan) to achieve single-cell suspension. Cells were then seeded at 70% confluency on dishes pre-coated with Matrigel and cultured in StemMACS iPS-Brew XF + 5 μM Y-27632 (72304) (STEMCELL Tech., Vancouver, BC, Canada). On day 1 (D1), the medium was replaced with neural induction medium (NIM) comprising the neural medium (NM) with SMAD (suppressor of mothers against decapentaplegic) inhibitors, + 0.5 μM LDN193189 (72147) (STEMCELL Tech.) + 10 μM SB431542 (Miltenyi Biotec), where NM is a mixture of 50% [*v*/*v*] DMEM: F12 + 50% [*v*/*v*] MACS Neuro Medium (130-093-570) (Miltenyi Biotec) + GlutaMax (35050061) (Thermo Fisher Scientific, Waltham, MA, USA) + NEAA (non-essential amino acids) (11140050) (Thermo Fisher Scientific) + N-2 (neuro-2) (17502048) (Thermo Fisher Scientific) + MACS NeuroBrew-21 (130-093-566) (Miltenyi Biotec). On D3, SHH (sonic hedgehog) inducers, 100 ng/mL SHH-C24II (130-095-723) (Miltenyi Biotec), 100 ng/mL FGF8b (fibroblast growth factor 8) (130-095-738) (Miltenyi Biotec), 2 μM Purmorphamine (130-104-465) (Miltenyi Biotec), and GSK3β (glycogen synthase kinase 3 beta) inhibitor, 3 μM CHIR99021 (130-104-172) (Miltenyi Biotec) was supplemented to the medium, which was then changed every 2–3 days until D10. At this point, stem cells have differentiated into iDA progenitors. These progenitor cells were cultured in NM + 100 ng/mL FGF8b + 20 ng/mL BDNF (bone-derived neurotrophic factor) (130-103-435) (Miltenyi Biotec) + 0.2 mM L-AA (L-ascorbic acid) (A5960) (Sigma, St. Louis, MO, USA), and the medium was changed every day. From D15, DA maturation medium, i.e., NM + 20 ng/mL GDNF (glia-derived neurotrophic factor) (130-108-986) (Miltenyi Biotec) + 20 ng/mL BDNF + 0.2 mM L-AA + 1 ng/mL TGFβ3 (transforming growth factor beta 3) (243-B3-002/CF) (R&D Systems, Minneapolis, MN, USA) + 0.5 mM db-cAMP (dibutyryl cyclic adenosine monophosphate) (D0260) (Sigma) + 10 μM DAPT (gamma secretase inhibitor) (2634) (Tocris Bio., Bristol, UK) was added and changed every other day until iDAs were ready on D40. Successful differentiation of iDAs was verified using gene expression assay (RT-qPCR) and protein expression assays (Western blotting, immunofluorescence, and flow cytometry) for various stem cell, neuronal, and dopaminergic markers.

### 2.4. Cellular ET Uptake and Liquid Chromatography Mass Spectrometry

ET was quantified as previously described [24]. Cells were washed thrice with ice-cold PBS (Thermo Fisher Scientific) before the addition of methanol spiked with a deuterated internal standard (ISTD) ET-d9. Next, samples were centrifuged at 20,000× *g* at 4 °C for 10 min, the supernatants collected in glass vials, and the contents evaporated at 37 °C under a N_2_ stream. Glass vials containing the sample residues were reconstituted in pure methanol and ET levels were analysed by liquid chromatography mass spectrometry (LC-MS/MS), using an Agilent 1290 UPLC system coupled with an Agilent 6460 ESI mass spectrometer (Agilent Technologies, Santa Clara, CA, USA). Samples were kept at 10 °C in the autosampler. 2 µL of the processed samples was injected into a Cogent Diamond-Hydride column (4 µm, 150 × 2.1 mm, 100 Å; MicroSolv Technology Corporation, Leland, NC, USA) maintained at 30 °C. Solvent A was acetonitrile in 0.1% formic acid, and Solvent B was 0.1% formic acid in ultrapure water. Chromatography was carried out at a flow rate of 0.5 mL/min using the following gradient: 1 min of 20% solvent B, followed by a 3 min gradient increase in solvent B to 40% to elute ET. The retention time for ET is 4.2 min.

Mass spectrometry was carried out using the positive ion, electrospray ionisation mode, with multiple reaction monitoring (MRM) for quantification of specific target ions. Capillary voltage was set at 3200 V, and the gas temperature was kept at 350 °C. Nitrogen sheath gas pressure for nebulising the sample was at 50 psi, and the gas flow was set at 12 L/min. Ultra-high purity nitrogen was used as the collision gas. Precursor to product ion transitions and fragmentor voltages (V)/collision energies (eV) for each compound were as follows: ET; 230.1 → 186, 103 V/9 eV and ET-d9; 239.1 → 195.1, 98 V/9 eV.

### 2.5. MTT Assay

A MTT Assay Kit (Sigma) was used to detect the impact of 6-OHDA on cellular metabolic activity. iDA cultures (D22) or SH-SY5Y cells were passaged and counted using a haemocytometer. One thousand cells were seeded per well in a 96-well plate and allowed to differentiate until day 40 prior to treatments. 15 μM 6-OHDA was found to induce 60–70% loss of cellular metabolic activity based on preliminary experiments. 1 mg of MTT was added into 1 mL of medium, which was incubated for 4 h at 37 °C. The medium in the plate was discarded after 4 h, and 200 µL DMSO (dimethylsulphoxide) was added to each well before being shaken for one minute for dissolution. Absorbance at 570 nm based on cellular dehydrogenase activity was measured in a microplate reader (Tecan, Switzerland). The different experimental conditions were normalised to control absorbance. Experiments were carried out three times.

### 2.6. Fluorescence Microscopy

Immunocytochemistry was employed to visualise OCTN1 expression, mitochondrial morphology, and mitochondrial ROS production. Cells were seeded onto 13 mm coverslips in 6-well plates prior to respective treatment conditions. For live staining, cells were washed twice with PBS before incubation with either 2.5 μM MitoSOX (Invitrogen^TM^, Waltham, MA, USA) or 50 nM of MitoTracker^TM^ Green FM (Thermo Fisher Scientific) for 15 min. Cells attached on the coverslips were washed thrice with PBS before removal. ProLong^TM^ Gold Antifade Mountant with DAPI (Thermo Fisher Scientific) was added before visualisation with a fluorescence microscope. For fixed staining, cells were washed twice with PBS before fixing with 3.7% paraformaldehyde (Thermo Fisher Scientific) for 20 min. Fixed cells were washed thrice with ultrapure water before permeabilisation with PBS containing 0.02% tween (Thermo Fisher Scientific) for 5 min. Blocking was done in blocking buffer comprising PBS, 0.02% tween, and 1% bovine serum albumin (BSA) (Thermo Fisher Scientific) for 1 h. After blocking, cells were incubated overnight (for at least 16 h) at 4 °C with primary antibody in blocking buffer. Mouse anti-neurofilament (#2F11) (Sigma), mouse anti-NeuN (LV1825845) (Millipore, Burlington, MA, USA), goat anti-beta tubulin III (MAB1195) (R&D Systems, Minneapolis, MN, USA) and goat anti-OCTN1 (sc-19819) (Santa Cruz, CA, USA) antibody were used at a 1:200 dilution. Expression of OCTN1 was further confirmed using Western blot after membrane extraction. APC-conjugated tyrosine hydroxylase (130-120-352) (Miltenyi Biotec) and FITC-conjugated tyrosine hydroxylase (130-120-350) (Miltenyi Biotec) were used at a 1:50 dilution. Binding of primary antibodies was followed by incubation with either anti-mouse secondary AF488 (green)-conjugated antibody (1:1000) (A28175) (Thermo Fisher Scientific), anti-goat secondary APC-conjugated antibody (1:1000) (A-865) (Thermo Fisher Scientific), anti-goat secondary AF647 (violet)-conjugated antibody (1:1000) (A-21447) (Thermo Fisher Scientific) in blocking buffer for 1 h at room temperature. Cells were washed thrice with PBS before the addition of ProLong^TM^ Gold Antifade Mountant with DAPI (Thermo Fisher Scientific) and visualisation with fluorescence microscope. Protein expression was visualised with Olympus FV3000 confocal microscope (Tokyo, Japan), and mROS production was visualised with Olympus DP70 fluorescence microscope (Tokyo, Japan).

### 2.7. Quantitative RT-PCR

TRIzol Reagent (Invitrogen^TM^) was used for RNA extraction as per the manufacturer’s instructions. cDNA was produced from reverse transcription of 1000 ng of RNA (High-Capacity cDNA Reverse Transcription Kit; Applied Biosystems, Waltham, MA, USA). Reverse transcription was performed in a T-Personal Thermocycler (Biometra, Germany) with conditions of 25 °C for 10 min, 37 °C for 30 min, followed by 85 °C for 5 min. qPCR was carried out to quantify *OCT4* (octamer-binding transcription factor 4), *NANOG* (NANOG homeobox), *MAP2* (microtubule associated protein 2), *NeuN* (neuronal nuclei), *NeuroD1* (neurogenic differentiation factor 1), *BDNF*, *FOXA2* (foxhead box protein A2) and *DAT* (dopamine transporter) mRNA expression, using SYBR Green Gene Expression Assay Probes (Applied Biosystems, USA) and SYBR Green Universal PCR Master Mix (Applied Biosystems, USA). *GAPDH* was used as the housekeeping gene. The RT-qPCR was performed in a 7500 Real-time PCR System (Applied Biosystems, USA) with conditions of 95 °C for 10 min, followed by 95 °C for 15 s and 60 °C for 1 min, for 40 cycles. Subsequently, the relative mRNA expression for the respective genes of interest was quantitated via the comparative CT (ΔΔCT) method. Primer sequences can be found in Supplementary Table S1.

### 2.8. Flow Cytometry

Flow cytometry measurements were performed using a CytoFlex LX flow cytometer (Beckman Coulter Life Sciences, Indianapolis, IN, USA), using 10^6^ cells per sample for analysis with 10,000 events per sample recorded. The FL1 channel was used to quantify cell death (propidium iodide, Ex/Em = 535/615 nm), free intracellular calcium (Fluo-4, Ex/Em = 488/525 nm), mitochondrial membrane potential (tetramethylrhodamine, methyl ester (TMRM), Ex/Em = 555/575 nm), mitochondrial ROS (MitoSOX, Ex/Em = 510/580 nm), and mitochondria (MitoTracker green Ex/Em = 490/526 nm). iDAs were cultured on coverslips washed with PBS before being stained with either propidium iodide, MitoSOX, or TMRM for 30 min. Cells were washed with PBS before 15 min fixation in 3.7% paraformaldehyde. Cells were permeabilised in perm buffer (Miltenyi Biotec) before blocking and primary antibody incubation of tyrosine hydroxylase that was FITC conjugated (130-120-350) (Miltenyi Biotec). Cells were then washed with PBS before analysis using flow cytometry. For flow cytometry data acquisition, fluorescent signals were measured on a logarithmic scale of four decades of log. Raw data were processed using FlowJo version 10.5.3.

### 2.9. ATP Assay

A commercially available ATP determination kit (Invitrogen^TM^) was used to study ATP levels in cells. Treated cells (iDA or SH-SY5Y cells) were collected and counted using a haemocytometer to ensure all samples contain equal number of cells (10^6^ cells). Upon centrifugation and removal of supernatant, 1 mL of boiling ultrapure water from an Arium pro^®^ ultrapure system was added into the cell pellet and incubated in a water bath for 10 min at 100 °C [55]. Samples were then cooled on ice for 30 s and supernatant utilised for ATP assay as per the manufacturer’s instructions. Luminescence readings of the samples were performed using Synergy H1 Microplate Reader (BioTek, Winooski, VT, USA). For all experiments, ATP standard curves were run in the range of 0.2 to 1.4 μM.

### 2.10. Western Blot

Cells were lysed with ProteoExtract^®^ Native Membrane Protein Extraction Kit (Millipore) for OCTN1 expression, performed as per the manufacturer’s instructions. Supernatant was collected after centrifugation at 13,000× *g* at 4 °C for 20 min. Protein concentration was determined using a bicinchoninic acid (BCA) protein assay kit (Sigma Aldrich). The samples were loaded and separated on precast SDS-polyacrylamide gels (Bio-Rad, Hercules, CA, USA). Proteins were electro-transferred to a nitrocellulose membrane (Bio-Rad) in transfer buffer containing 48 mmol/L Tris-HCl, 39 mmol/L glycine, 0.037% SDS, and 20% methanol at 4 °C for 1 h. Blocking was done in 2.5% non-fat milk for 1 h at room temperature. Binding of OCTN1 primary goat anti-OCTN1 (sc-19819) (Santa Cruz, CA, USA) antibody at 1:200 dilution, overnight at 4 °C, was followed by incubation with secondary horseradish peroxidase-conjugated IgG in 2.5% non-fat milk for 1 h at room temperature. Anti-mouse IgG HRP were obtained from Sigma Aldrich and dilution of 1:5000 was used. The blots were visualised with SuperSignal^TM^ West Femto Maximum Sensitivity Substrate (Thermo Fisher Scientific) using iBright Imaging systems (Invitrogen^TM^). The blots were processed using open access software platform FIJI version 1.53r (ImageJ).

### 2.11. Protein Carbonylation Assay

Cells were lysed with Pierce^®^ RIPA Lysis and Extraction buffer (Thermo Fisher Scientific) for protein carbonylation assay. Supernatant was collected after centrifugation at 13,000× *g* at 4 °C for 20 min. Protein concentration was determined using a bicinchoninic acid (BCA) protein assay kit (Sigma Aldrich). OxyBlot Protein Oxidation Detection Kit (Chemicon/Millipore, Temecula, CA, USA) was utilised. The samples were derivatised and prepared as per the manufacturer’s instructions prior to being loaded and separated on precast SDS-polyacrylamide gels (Bio-Rad). Proteins were electro-transferred to a nitrocellulose membrane (Bio-Rad) in transfer buffer containing 48 mmol/L Tris-HCl, 39 mmol/L glycine, 0.037% SDS, and 20% methanol at 4 °C for 1 h. Blocking, incubation with 2,4-Dinitrophenylhydrazine (DNPH) antibody and secondary antibody, respectively, were also performed as specified by the manufacturer. Binding of primary antibodies was followed by incubation with secondary horseradish peroxidase (HRP) conjugated IgG in 2.5% non-fat milk for 1 h at room temperature. Anti-mouse IgG HRP was obtained from Sigma Aldrich, and a dilution of 1:5000 was used. The blots were visualised with SuperSignal^TM^ West Femto Maximum Sensitivity Substrate (Thermo Fisher Scientific) using iBright Imaging systems (Invitrogen^TM^). The blots were processed using open access software platform FIJI (ImageJ).

### 2.12. Tyrosine Hydroxylase Protein Assay

Flow cytometry was employed to assess tyrosine hydroxylase (TH) protein expression in SH-SY5Y cells. Cells were first detached, and cell pellets were washed with PBS. Cells were incubated for 30 min with 3.7% paraformaldehyde before the permeabilisation step for another 30 min. Blocking was done with 5% BSA before 30 min incubation with FITC-conjugated TH antibody and APC-conjugated Tau antibody (130-119-363) (Miltenyi Biotec). The tau neuronal marker serves as the housekeeping protein. Flow cytometry measurements were performed on a Cytoflex LX flow cytometer (Beckman Coulter Life Sciences), using 1,000,000 cells per sample for analysis. In total, 10,000 events per sample were recorded. Fluorescent signals were measured on a logarithmic scale of four decades of log. Raw data were processed using FlowJo version 10.5.3.

### 2.13. Dopamine Detection ELISA Kit

Dopamine levels were quantified using a commercially available dopamine detection kit (Novus Biologicals, Littleton, CO, USA). The colorimetric assay is a 90 min, single-wash sandwich ELISA designed for quantitative measurement of dopamine. Treated D40 iDA cultures in 12-well plates have 500 μL of total conditioned media collected for dopamine analysis. The same treated cells (with conditioned media removed) were lysed and protein concentration was quantified using a Pierce BCA protein assay kit. Conditioned media from treated cells were collected and centrifuged to remove cellular debris. Protein concentrations in all conditioned media were normalised based on the protein concentration of lysed cells using sample dilution buffer. Next, 50 μL of diluted sample (normalised) and 50 μL of antibody cocktail were added to each well, followed by 1 h incubation on a shaker at 400 rpm. After 1 h, the 3,3′,5,5′-Tetramethylbenzidine (TMB) solution provided was added before the addition of stop solution as per the manufacturer’s instructions. Dopamine standards were prepared as well. Absorbance readings at 450 nm of the samples were performed using Synergy H1 Microplate Reader (BioTek, USA). For all experiments, dopamine standard curves were run in the range of 2.19 to 140 ng/mL.

### 2.14. Statistical Analysis

Data are shown as mean ± SD (standard deviation). All analysis was performed using GraphPad prism 9.0 software. Comparisons were conducted by one-way ANOVAs, with Bonferroni correction for multiple comparison tests. *p* < 0.05 was regarded as statistically significant.

## 3. Results

### 3.1. Generation of hiPSC-Derived Day 40 Dopaminergic Neurons

**Figure 1:**
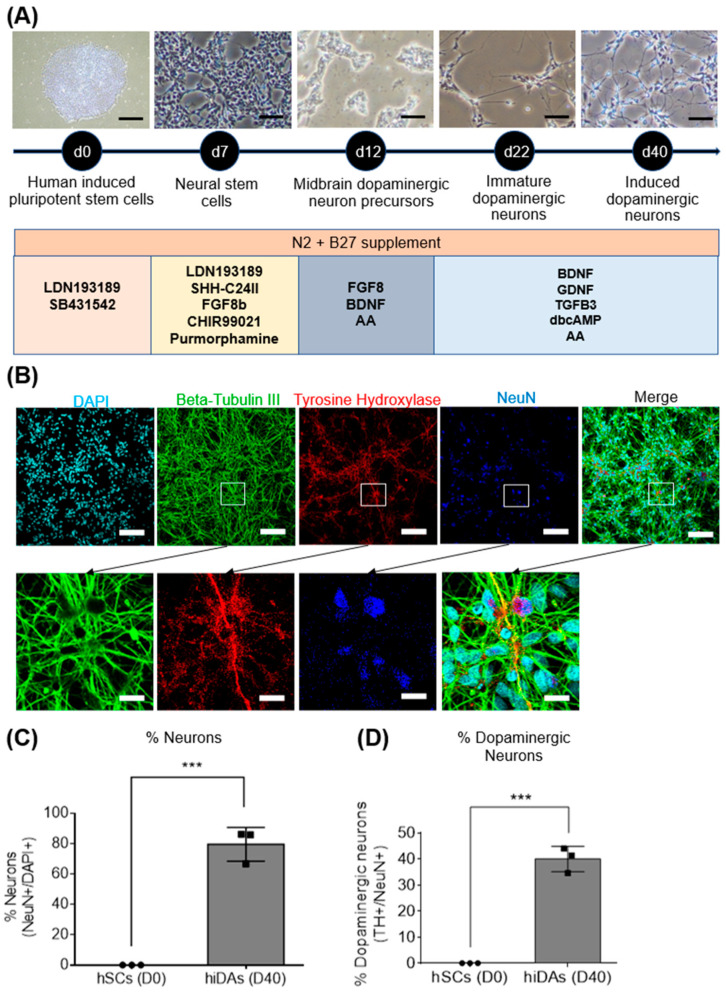
Derivation of day 40 dopaminergic neurons and quality control analysis. (**A**) Schematic representation of differentiation protocol to derive day-40-induced dopaminergic neurons from hiPSCs (BJ and GM23720) and hESC (H9). Scale bar= 100 µm (**B**) Confocal imaging of neuronal and dopaminergic markers. DAPI stains the nucleus. Beta-Tubulin III and NeuN are neuronal markers. Tyrosine hydroxylase (TH) is a dopaminergic marker. Scale bar = 100 µm; Scale bar for zoomed in figures = 20 µm (**C**,**D**) ImageJ analysis of confocal images of TH and NeuN to quantify the percentage of neurons and dopaminergic neurons present in the culture. Data are represented as mean ± SD (*n* = 3). Data were analysed by unpaired Student’s *t*-test. *** *p* ≤ 0.001.

We differentiated human induced pluripotent stem cells (hiPSCs; BJ and GM23720) and human embryonic stem cells (hESC; H9) to investigate the protective effect of ET on 6-OHDA-treated induced human dopaminergic neurons, a model highly relevant to human PD. We adopted a 40-day differentiation protocol, using various small molecules and growth factors (Figure 1A). Results show a significant decrease in the pluripotency markers OCT4 and NANOG (Supplementary Figure S1A) but significant increases in the neuronal markers *MAP2*, *NeuN*, *BDNF* and *NeuroD1* (Supplementary Figure S1B) and the dopaminergic-specific markers *FOXA2* and *DAT* (Supplementary Figure S1C). Protein expression of neuronal and dopaminergic markers was also performed using confocal imaging, which showed that our day 40 iDAs express neuronal proteins of NeuN and TUBB3, and dopaminergic neuron-specific marker TH (Figure 1B). Neuronal yield and dopaminergic yield were found to be approximately 80% (Figure 1C) and 40% (Figure 1D), respectively, which is consistent with other studies [56,57].

### 3.2. ET Protects Day 40 iDA Cultures against 6-OHDA-Induced Increase in mROS, Loss of MMP, Reduction in ATP Levels, and Loss of Dopamine Secretion

**Figure 2:**
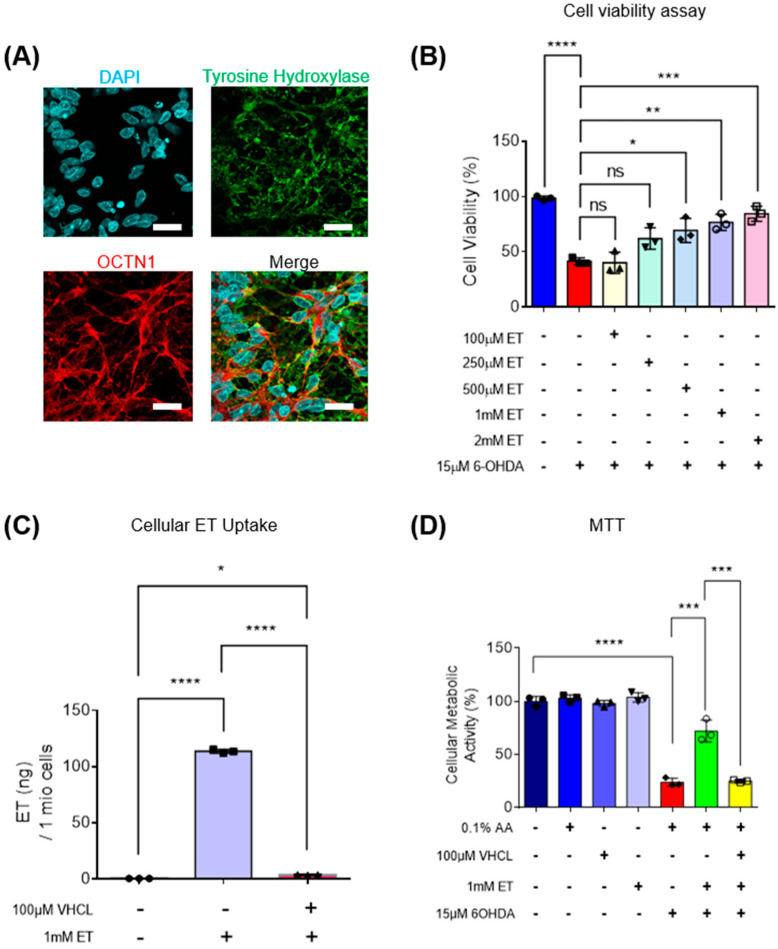
Intracellular uptake of ET, mediated through OCTN1 transporters, protects iDA cultures against 6-OHDA-induced cell death in a concentration-dependent manner. (**A**) Confocal imaging showing expression of TH and OCTN1 in D40 iDAs. Images are representative of three independent experiments (*n* = 3). Scale bar = 20 µm (**B**) Protection of ET against 6-OHDA neurotoxicity was dose-dependent. (**C**) LC-MS assay showing ET uptake into day 40 iDAs (*n* = 3). (**D**) MTT assay showed that 6-OHDA induced a 70–80% loss of metabolic activity in iDA cultures. Co-treatment with a non-specific inhibitor of OCTN1, verapamil hydrochloride (VHCL), abrogated the protective effects of ET. (Control: no treatment; 6OHDA: 15 µM 6-hydroxydopamine; ET: 1 mM ergothioneine; VHCL: 100 µM verapamil hydrochloride). Data were analysed by one-way ANOVA with Bonferroni’s multiple comparison post hoc test. ns: non-significant, * *p* ≤ 0.05. ** *p* ≤ 0.01. *** *p* ≤ 0.001. **** *p* ≤ 0.0001.

**Figure 3:**
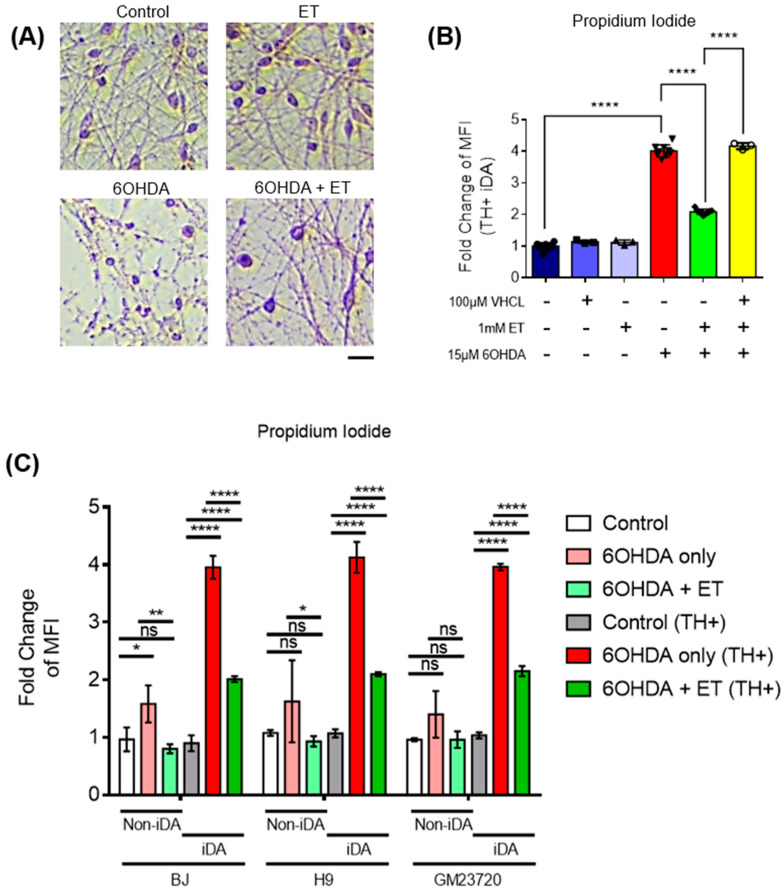
ET-mediated protection of 6-OHDA-treated iDAs and non-iDAs in culture. (**A**) Haematoxylin and eosin (H&E) staining to show changes of neuronal morphology from various treatment groups. Scale bar = 20 µm (**B**) Propidium iodide assay using flow cytometry showing changes in quantity of non-viable TH-positive (TH+) cells. Higher MFI readings denote an increase in non-viable TH+ cells from the treatment group and vice versa. (**C**) Comparison of cell death between iDAs (TH+ neurons) and non-iDAs (TH- neurons) with or without ET. (**B**,**C**) Data are represented as mean ± SD (*n* = 3). (Control: no treatment; 6OHDA: 15 µM 6-hydroxydopamine; ET: 1 mM ergothioneine; VHCL: 100 µM verapamil hydrochloride). Data were analysed by one-way ANOVA with Bonferroni’s multiple comparison post hoc test. ns: non-significant, * *p* ≤ 0.05. ** *p* ≤ 0.01. **** *p* ≤ 0.0001.

**Figure 4:**
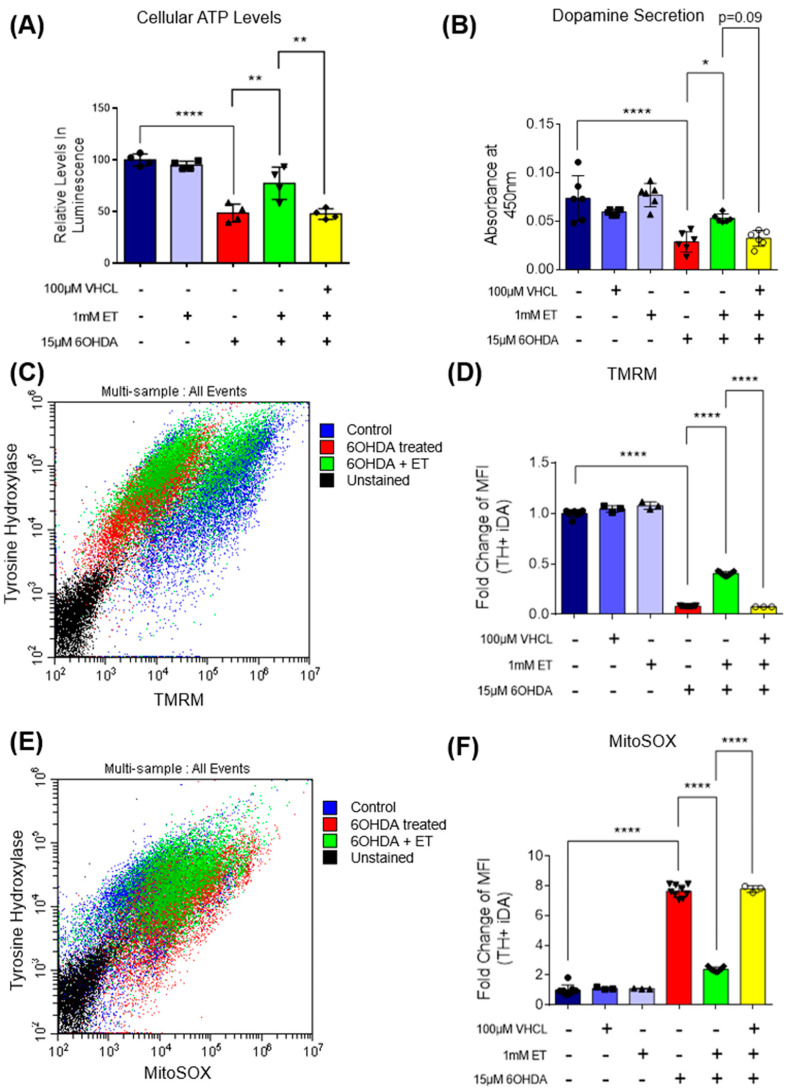
Effect of ET on intracellular ATP levels and dopamine secretion in 6-OHDA-treated iDA cultures; TH+ Day 40 iDAs were also analysed for mitochondrial ROS and mitochondrial membrane potential levels. (**A**) ATP assay showing relative changes in intracellular ATP levels. Higher absorbance values denote greater quantities of ATP in the cell lysates of the treatment group. (**B**) Dopamine secretion assay showing the changes in the amounts of dopamine being secreted by the day 40 iDAs in each treatment group. Higher absorbance values denote greater quantities of dopamine. (**C**) Scatter plot of flow cytometry data (TH-TMRM co-staining) of the day 40 iDAs from the different treatment groups. (**D**) Bar chart of flow cytometry data for TH and TMRM co-staining to show relative changes in the different treatment groups. (**E**) Scatter plot of flow cytometry data (TH-MitoSOX co-staining) of the day 40 iDAs from the different treatment groups. (**F**) Bar chart of flow cytometry data for TH and MitoSOX co-staining to show relative changes in the different treatment groups. (**A**,**B**,**D**,**F**) Data are represented as mean ± SD (*n* = 3). (Control: no treatment; 6OHDA: 15 µM 6-hydroxydopamine; ET: 1 mM ergothioneine; VHCL: 100 µM verapamil hydrochloride). Data were analysed by one-way ANOVA with Bonferroni’s multiple comparison post hoc test. * *p* ≤ 0.05. ** *p* ≤ 0.01. **** *p* ≤ 0.0001.

**Figure 5:**
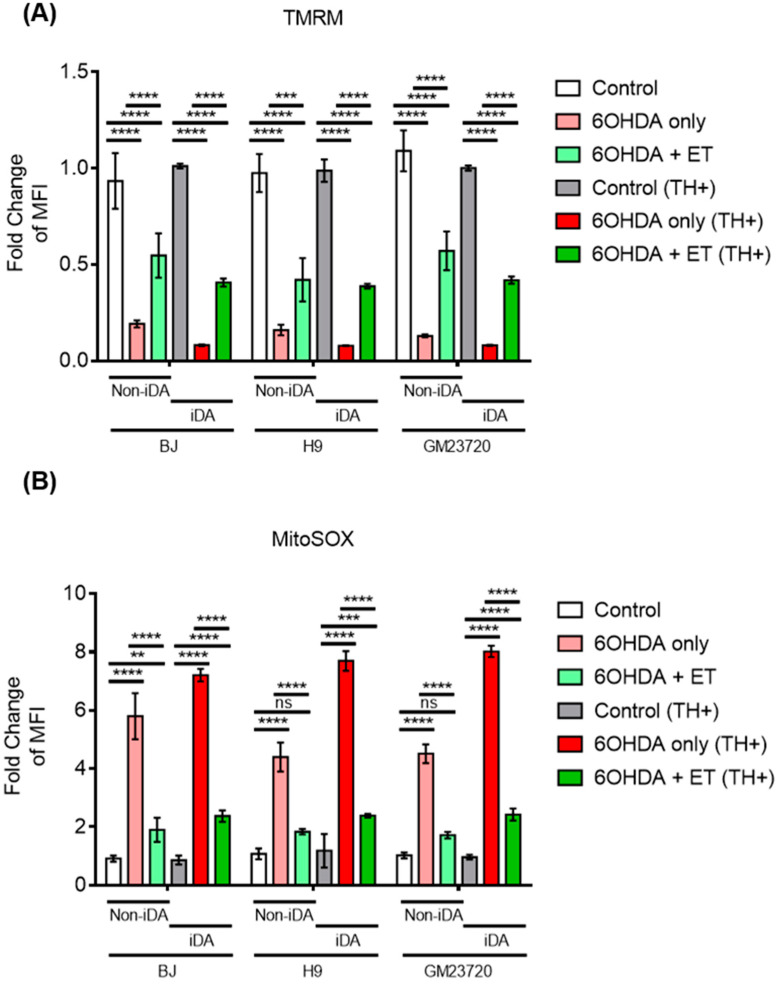
Effect of ET and 6OHDA on mitochondrial membrane potential and mitochondrial ROS in both non-iDAs and TH+ iDAs population. (**A**) Comparison of the effect of 6-OHDA on mitochondrial membrane potential between iDAs (TH+ neurons) and non-iDAs (TH- neurons) with or without ET. (**B**) Comparison of the effect of 6-OHDA on mitochondrial ROS levels between iDAs and non-iDAs with or without ET. (**A**,**B**) Data are represented as mean ± SD (*n* = 3). (Control: no treatment; 6OHDA: 15 µM 6-hydroxydopamine; ET: 1 mM ergothioneine). Data were analysed by one-way ANOVA with Bonferroni’s multiple comparison post hoc test. ns: non-significant, ** *p* ≤ 0.01. *** *p* ≤ 0.001. **** *p* ≤ 0.0001.

MTT assay showed that 6-OHDA induced a 70–80% loss of metabolic activity in iDA cultures (Figure 2D). 6-OHDA was also found to induce neuronal degeneration observed under the microscope (Figure 3A). Degenerating neurons appear fragmented with neurite degeneration, similar to other in vitro neuronal degeneration phenotypes [58]. 6-OHDA treatment led to a significant increase in non-viable cells. iDA cultures express the OCTN1 transporter that mediates cellular ET uptake (Figure 2A). Protection of iDA cultures by ET against loss of viability induced by 6-OHDA is concentration dependent (Figure 2B). 1 mM of ET was used in subsequent experiments as it is similar to achievable cellular levels [59,60]. The non-specific inhibitor of OCTN1, verapamil hydrochloride (VHCL), greatly reduced intracellular ET levels (Figure 2C). 6-OHDA resulted in a higher proportion of non-viable cells in TH-positive (TH+) iDAs than TH-negative (TH−) non-iDAs (Figure 3C). In both TH+ and TH- populations, ET significantly ameliorated the 6-OHDA-induced increase in non-viable cells (except GM23720 non-iDAs; *p* = 0.233) (Figure 3C). 6-OHDA treatment also caused a decrease in intracellular ATP levels, while co-treatment with ET was able to attenuate the decrease (Figure 4A). Dopamine secretion assay shows that 6-OHDA caused a significant reduction in the amount of dopamine secreted by day 40 iDA cultures, while co-treatment with ET was able to attenuate the decrease (Figure 4B). We also investigated the effects of 6-OHDA on mitochondrial function, as measured by the TMRM (mitochondrial membrane potential—MMP) and MitoSOX (mitochondrial ROS) probes. Murphy et al. [61] discussed the pros and cons of this method, and results must always be interpreted with caution, e.g., they can be affected by changes in mitochondrial size, shape, and membrane potential. From the results, we found that 6-OHDA caused a significant decrease in MMP (Figure 4C,D and Figure 5A) and an apparent significant increase in mROS levels (Figure 4E,F and Figure 5B) in both iDAs and non-iDAs. The effect of 6-OHDA on mitochondrial membrane potential and mitochondrial ROS levels was more significant in iDAs than non-iDAs (Figure 5A,B). VHCL abrogated the effects of ET.

### 3.3. ET Uptake Also Protects TH+ SH-SY5Y Neuron-like Cells against 6-OHDA Neurotoxicity

**Figure 6:**
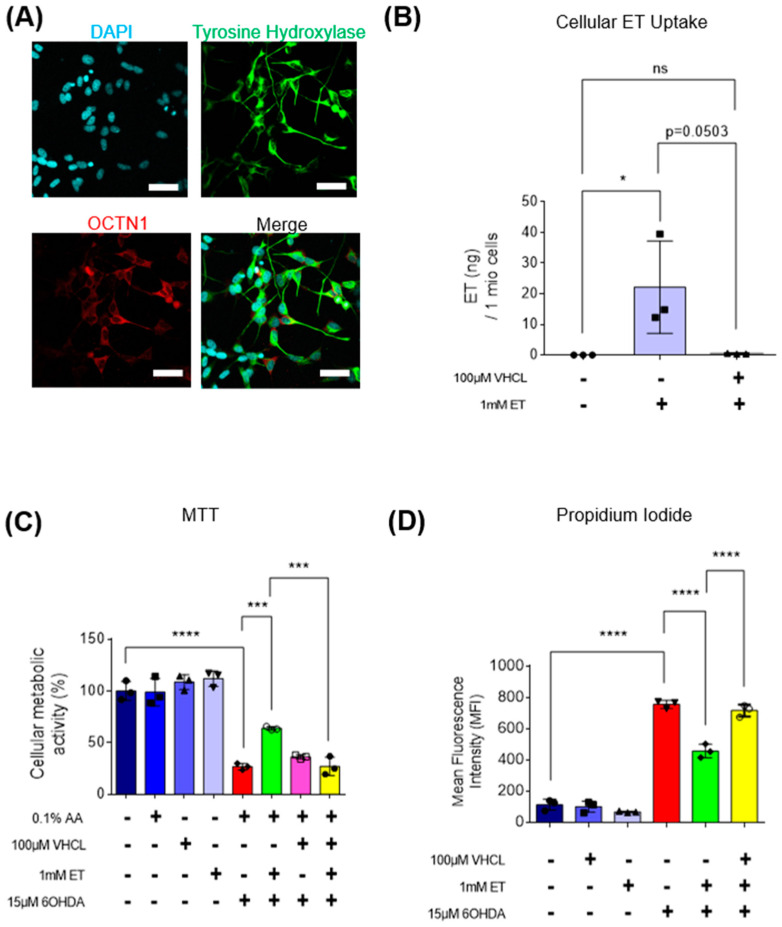
Intracellular uptake of ET, mediated through OCTN1 transporters, also protects TH+ SH-SY5Y cultures against 6-OHDA-induced cell death. (**A**) Confocal imaging showing expression of TH and OCTN1 in SH-SY5Y cells. Images are representative of three independent experiments (*n* = 3). Scale bar = 50 µm (**B**) LC-MS assay showing ET uptake into day 40 iDAs (*n* = 3). (**C**) MTT assay showed that 6-OHDA induced a 70–80% loss of metabolic activity in iDA cultures. (**D**) Propidium iodide assay using flow cytometry showing changes in quantity of non-viable cells. Higher MFI readings denote an increase in non-viable cells from the treatment group and vice versa. (**C**,**D**) Co-treatment with a non-specific inhibitor of OCTN1, verapamil hydrochloride (VHCL), largely abrogated the protective effects of ET. (**B**–**D**) Data are represented as mean ± SD (*n* = 3). (Control: no treatment; 6OHDA: 15 µM 6-hydroxydopamine; ET: 1 mM ergothioneine; VHCL: 100 µM verapamil hydrochloride). Data were analysed by one-way ANOVA with Bonferroni’s multiple comparison post hoc test. ns: non-significant, * *p* ≤ 0.05. *** *p* ≤ 0.001. **** *p* ≤ 0.0001.

**Figure 7:**
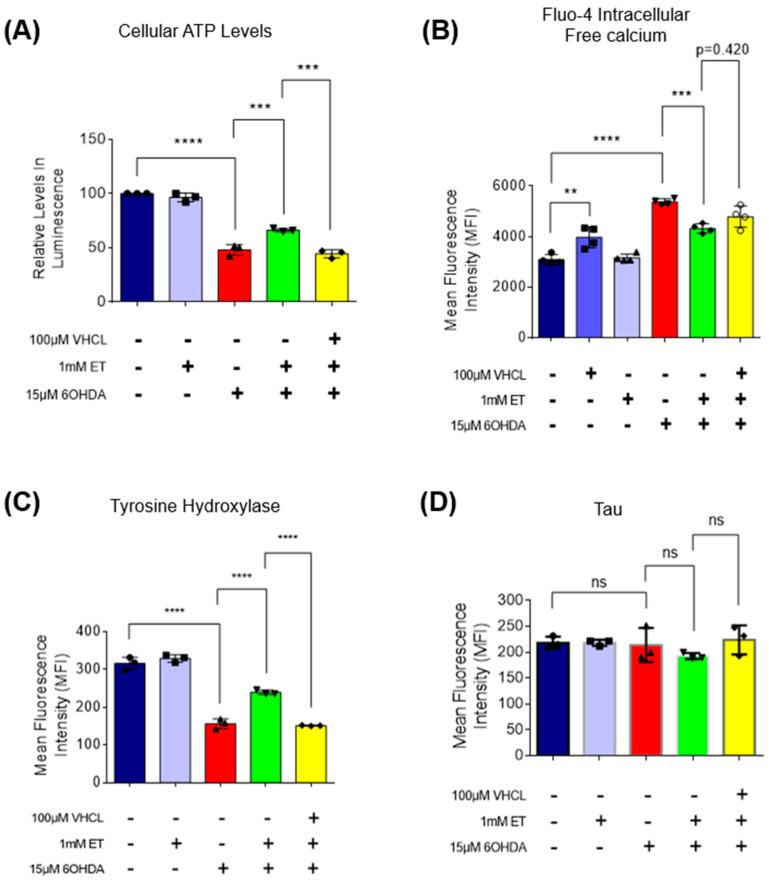
Effect of ET on cellular metabolism and tyrosine hydroxylase levels in 6-OHDA-treated SH-SY5Y cells. (**A**) ATP assay showing relative levels in intracellular ATP as compared to the control group. (**B**) Fluo-4 AM assay measures intracellular free calcium levels. Data are represented as mean ± SD (*n* = 4). (**C**) Flow cytometry analysis to quantify relative amounts of tyrosine hydroxylase (TH) protein expression as compared to control. (**D**) Flow cytometry analysis to quantify relative tau neuronal protein expression among the treatment groups as compared to control. (**A**–**D**) (Control: no treatment; 6OHDA: 15 µM 6-hydroxydopamine; ET: 1 mM ergothioneine; VHCL: 100 µM verapamil hydrochloride). Data were analysed by one-way ANOVA with Bonferroni’s multiple comparison post hoc test. Unless stated in figures, ns: non-significant, ** *p* ≤ 0.01, *** *p* ≤ 0.001, **** *p* ≤ 0.0001.

**Figure 8:**
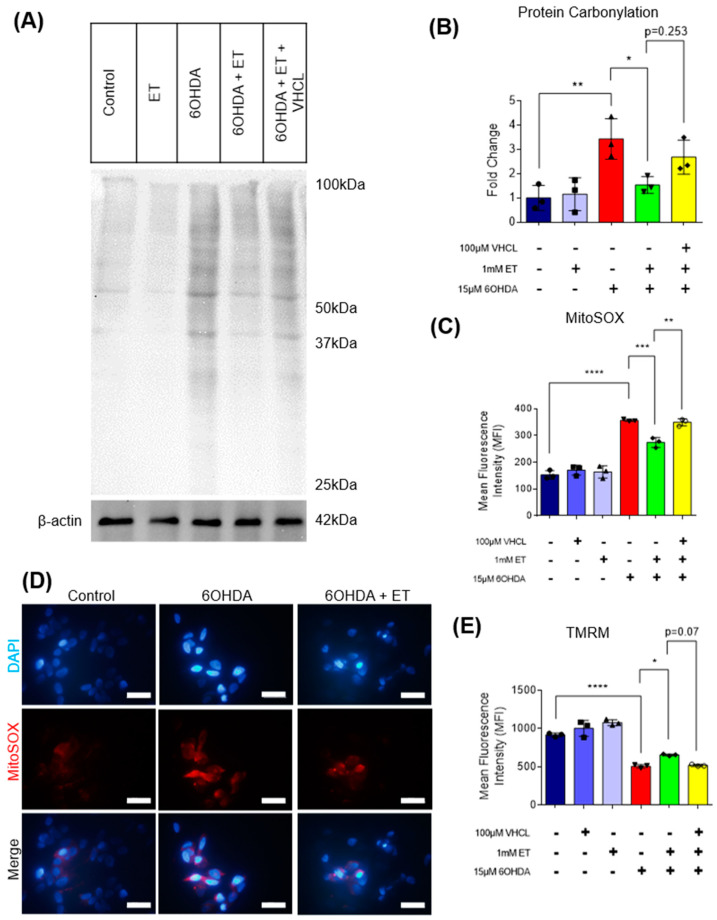
Effect of intracellular ET on oxidative stress and mitochondrial membrane potential in 6-OHDA-treated SH-SY5Y cells. (**A**) Protein carbonylation immunoblot of the different treatment groups. β-Actin was used as the housekeeping protein for normalisation. (**B**) ImageJ analysis of immunoblot to quantify relative amounts of oxidised proteins between the treatment groups, as compared to control. (**C**) MitoSOX assay using flow cytometry showing changes in mROS levels. Data are represented as mean ± SD (*n* = 3). (**D**) Fluorescence cytochemistry live-cell imaging for mROS. DAPI (blue) stains for nucleus, whereas MitoSOX (red) stains for mROS. Scale bar = 50 µm (**E**) TMRM assay measures mitochondrial membrane potential (MMP). (Control: no treatment; 6OHDA: 15 µM 6-hydroxydopamine; ET: 1 mM ergothioneine; VHCL: 100 µM verapamil hydrochloride). Data are represented as mean ± SD (*n* = 3). Data were analysed by one-way ANOVA with Bonferroni’s multiple comparison post hoc test. Unless stated in figures, * *p* ≤ 0.05, ** *p* ≤ 0.01, *** *p* ≤ 0.001, **** *p* ≤ 0.0001.

To verify if ET can protect other TH+ cells from the neurotoxicity of 6-OHDA, the SH-SY5Y human neuroblastoma cell line, a well-accepted model in PD studies [51], was also used. SH-SY5Y cells also express TH and the OCTN1 transporter (Figure 6A); uptake of ET was abolished with coincubation of VHCL (Figure 6B). 6-OHDA-treated SH-SY5Y cells revealed a 60–70% loss of metabolic activity (Figure 6C) and a significant increase in non-viable cells (Figure 6D). 6-OHDA also significantly affected cellular metabolism as evidenced by the reduction in intracellular ATP levels (Figure 7A), increased intracellular free calcium (Figure 7B), decreased mitochondrial membrane potential (MMP) (Figure 8E), and increased mitochondrial ROS (mROS) levels (Figure 8C,D), as measured by the MitoSOX probe. Since heightened levels of ROS can promote oxidative protein damage, as revealed by protein carbonyl formation [62], and increased brain protein carbonyls is a feature of PD [8], we performed a protein carbonyl assay by immunoblotting. 6-OHDA treatment led to a significant increase in oxidised proteins (approximately 3.5-fold) as compared to the control (Figure 8A,B). Tyrosine hydroxylase (TH) was also investigated. Using flow cytometry, 6-OHDA treatment significantly reduced TH protein levels as compared to the control (Figure 7C), and 6-OHDA decreased dopamine secretion in day 40 iDAs. Tau protein, which is also implicated in PD [63], did not appear to have its levels affected by either 6-OHDA or ET treatment (Figure 7D). Co-treatment with ET was able to attenuate any changes induced by 6-OHDA. The addition of VHCL tended to abrogate (note *p* values on the figure) the effects of ET, although the results were not always significant (*p* > 0.05). These findings are consistent with our results involving D40 iDAs.

## 4. Discussion

In this study, we investigated the effect of ET on cell death caused by 6-OHDA in an iPSC model of PD. 6-OHDA-induced oxidative stress occurs as early as after 4 h of incubation, but cell death occurs within 24 h in rat [64], mouse [46], and human [65] dopaminergic cells. A concentration-dependent decrease in cellular metabolic activity with 60–70% loss of activity was observed at 15 μM 6-OHDA concentration with 24 h incubation. This concentration of 6-OHDA was selected for further experiments, while 1 mM of ET was used on the basis of preliminary data [59,60]. Dose-dependent ET protection of D40 iDA cultures against 6-OHDA is presented in Figure 2B. Ergothioneine (ET) is a cytoprotective compound that accumulates at high levels in tissues during times of oxidative damage [22,66]. LC-MS analysis confirmed that verapamil hydrochloride (VHCL), a non-specific inhibitor of OCTN1, reduced intracellular ET levels (Figure 2C and Figure 6B) and decreased the protective effect of ET. This demonstrated that the protective effects of ET observed in our experiments are largely or entirely dependent on cellular uptake of ET and not through, perhaps, extracellular neutralisation of 6-OHDA by direct reaction of ET with it or its oxidation products. Previous studies have found that OCTN1 is important for cellular uptake of ET in endothelial cells [67] and that OCTN1 levels could be elevated in response to tissue injury, e.g., in fatty liver [68] or kidney disease [69]. To demonstrate that OCTN1 is present in the cells, Western blot (Supplementary Figure S2) and fluorescence imaging (Figure 2A and Figure 6A) were carried out, but it should be noted that there is considerable variability in the specificity of commercial antibodies for OCTN1; nevertheless, our direct measurements of ET showed that it entered the cells and VHCL prevented that. Tyrosine hydroxylase (an enzyme involved in the production of dopamine) was expressed in both D40 iDAs (Figure 1B and Figure 2A) and SH-SY5Y cells (Figure 6A). Results showed that ET could protect against 6-OHDA-induced degeneration of both human iDAs and SH-SY5Y cells. VHCL abolished the protective effect of ET on cell viability.

Mitochondrial dysfunction was also ameliorated by ET in 6-OHDA treated iDA (TH+) and non-iDA neurons (TH-). Mitochondrial dysfunction is characterised by a decrease in ΔΨm, reduced ATP generation, and an increase in mitochondrial ROS (mROS) production [70]. 6-OHDA is thought to induce mitochondrial dysfunction in part through the inhibition of complex I of the mitochondrial electron transport chain, which contributes to a decreased ΔΨm, reduced ATP generation, increase in ROS production, and apoptotic cell death [48]. Similar to other studies [71,72], we found that 6-OHDA induced mitochondrial dysfunction as evidenced by a reduction of ΔΨm, decreased cellular ATP levels and increased mROS levels. ET ameliorated the effects of 6-OHDA with a smaller decrease in ΔΨm and cellular ATP levels and a lower increase in mROS levels. VHCL inhibited the protective effect of ET on mitochondrial dysfunction. Our results add to previous findings that ET has neuroprotective functions [21,26,28,43] which could potentially aid in the prevention and treatment of PD. We would also like to acknowledge the paper that was published by Yuzawa et al. (2024) [43] while we were preparing this manuscript. Yuzawa et al. reported the protective effect of ET against 6-OHDA neurotoxin in immortalised mouse hypothalamic cells (GT-17), a cell line perhaps less relevant to PD. Nevertheless, their data further illustrate the potential protective effect of ET against neurodegeneration.

The protective effect of ET against 6-OHDA was also observed in TH+ SHSY5Y cells. Besides loss of cell viability, treatment of SH-SY5Y cells with 15 μM 6-OHDA resulted in a significant increase in intracellular free calcium levels. Increases in intracellular free calcium levels are known to induce mitochondrial oxidative stress-mediated apoptosis [73]. ET reduced the 6-OHDA-induced increase in intracellular calcium levels. The reduced intracellular calcium levels could be due to an effect of ET as a cytoprotectant or chelator of divalent metal ions, including Ca^2+^ [74]. Our results demonstrate that ET could aid in the maintenance of intracellular calcium homeostasis. Treatment of SH-SY5Y cells with 15 μM 6-OHDA also resulted in a significant increase in total carbonylated proteins. Protein carbonylation is increased in the brains of PD patients [8,75] and in cells that are undergoing oxidative stress [76]. Oxidised proteins contribute to increased ER stress and increased unfolded protein response (UPR) [77] and can result in cellular apoptosis [78,79]. 6-OHDA was found to induce an increase in carbonylated proteins in SH-SY5Y cells [80], PC12 cells [81], and rats [82]. Results indicate a protective effect of ET against protein carbonyl formation. Moreover, ET was able to ameliorate the 6-OHDA-induced decrease in tyrosine hydroxylase levels. Tyrosine hydroxylase (TH) is the rate-limiting enzyme in dopamine production, and low dopamine levels play a key role in PD pathogenesis [83,84]. Similar to our study on SH-SY5Y cells, 6-OHDA treated rats were found to have reduced tyrosine hydroxylase [85]. The effect of ET on restoring TH levels could be clinically relevant since PD is caused by loss of dopamine-producing neurons. The pathology of PD involves mitochondrial dysfunction, oxidative stress, neuroinflammation, and aberrant protein homeostasis [86]. Besides the protective effect of ET as an anti-oxidant and improving cellular bioenergetics, ET could have cytoprotective effects through its influence on neurogenesis [87], potential epigenetic modifications [88], and regulation of sirtuins, a family of NAD^+^-dependent deacetylases [89,90]. Our work demonstrates mitochondrial protection by ET. Moreover, effects of ET on neuroinflammation and aberrant protein homeostasis contributing to PD can be further studied.

In summary, our findings demonstrate that ET can protect human iDA and non-iDA neurons against a 6-OHDA-induced increase in cell death and metabolic dysfunction. Results also suggest that ET has disease-modifying potential by increasing dopamine levels secreted by iDA neurons. Protective effects of ET were abrogated when cell cultures were cotreated with VHCL, a non-specific inhibitor of OCTN1, the ET transporter, suggesting that ET uptake into cells is necessary for protection against 6-OHDA. Results in SH-SY5Y cells also demonstrated the protective effects of ET against a 6-OHDA-induced increase in intracellular free calcium, increase in total carbonylated proteins, and reduction in tyrosine hydroxylase levels. In this paper, we have also shown the protective effects of ET against mitochondrial dysfunction. Uptake of ET into mitochondria in vivo due to conflicting data with regard to the localisation of OCTN1 on mitochondria is unclear [39,91,92]. Further studies also need to be carried out to validate the protective effect of ET in animal models of PD.

## Data Availability

The data are available upon request.

## Supplementary Materials

The following supporting information can be downloaded at https://www.mdpi.com/article/10.3390/antiox13060693/s1. Figure S1: qPCR quality control of day 40 dopaminergic neurons; Figure S2: Western blot showing expression of OCTN1 in SH-SY5Y, D40 iDA and 6OHDA treated D40 iDA lysates, respectively; Table S1: Primer sequences used for RT-qPCR.
