# Supplementary material for: Ergothioneine-Mediated Neuroprotection of Human iPSC-Derived Dopaminergic Neurons
Source: Antioxidants (Basel). 2024 Jun 5;13(6):693. doi: 10.3390/antiox13060693 (PMC11200999; doi:10.3390/antiox13060693)
Supplement: Supplementary file 1 [file antioxidants-13-00693-s001.zip › antioxidants-3018502-supplementary.pdf]

# Supplementary Materials

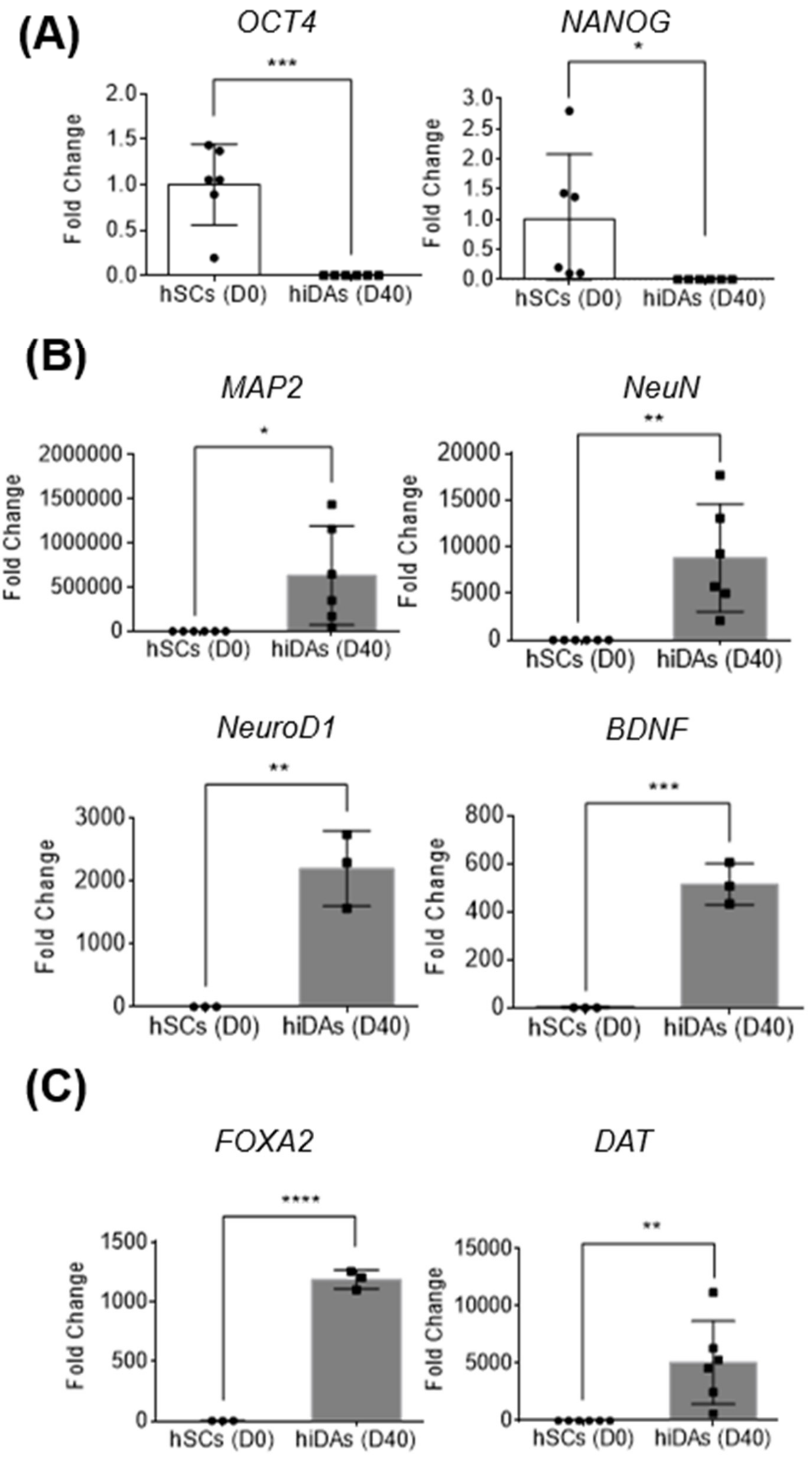


**Supplementary Figure S1. qPCR quality control of day 40 dopaminergic neurons.** (**A**) RT-qPCR analysis of relative mRNA expression of stem cell markers *OCT4* and *NANOG*. (**B**) RT-qPCR analysis of relative mRNA expression of neuronal markers *MAP2*, *NeuN*, *NeuroD1* and *BDNF*. (**C**) RT-qPCR analysis of relative mRNA expression of dopaminergic neuron-specific markers *FOXA2* and *DAT*. (**A**–**C**) Data is represented as

mean ± SD ($n \geq 3$). Data was analyzed by unpaired student's t-test. Data was analyzed by unpaired student's t-test. * $p \leq 0.05$. ** $p \leq 0.01$. *** $p \leq 0.001$. **** $p \leq 0.0001$.

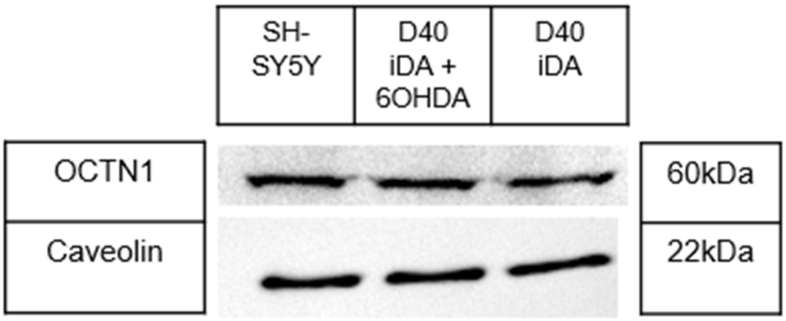


**Supplementary Figure S2. Western blot showing expression of OCTN1 in SH-SY5Y, D40 iDA and 6OHDA treated D40 iDA lysates, respectively**. Images are representative of three independent experiments (n = 3).

**Supplementary Table S1.** Primer sequences used for RT-qPCR.

| S/N | Primers (*Homo Sapiens*) | Gene accession number | Sequences (5′ to 3′) |
|---|---|---|---|
| 1 | *GAPDH* | NM_002046 | Forward: GCAAATTCCATGGCACCGT Reverse: GCCCCACTTGATTTTGGAGG |
| 2 | *ACTB* | NM_001101 | Forward: AGATGAGATTGGCATGGCTTTA Reverse: GGACTTCCTGTAACAACGCATC |
| 3 | *OCT4* | NM_002701 | Forward: CCTGAAGCAGAAGAGGATCACC Reverse: AAAGCGGCAGATGGTCGTTTGG |
| 4 | *NANOG* | NM_024865 | Forward: CTCCAACATCCTGAACCTCAGC Reverse: CGTCACACCATTGCTATTCTTCG |
| 5 | *MAP2* | NM_002374 | Forward: AGGCTGTAGCAGTCCTGAAAGG Reverse: CTTCCTCCACTGTGACAGTCTG |
| 6 | *NeuN* | NM_001082575 | Forward: TACGCAGCCTACAGATACGCTC Reverse: TGGTTCCAATGCTGTAGGTCGC |
| 7 | *NeuroD1* | NM_002500 | Forward: GGTGCCTTGCTATTCTAAGACGC Reverse: GCAAAGCGTCTGAACGAAGGAG |
| 8 | *BDNF* | NM_170734 | Forward: CATCCGAGGACAAGGTGGCTTG Reverse: GCCGAACTTTCTGGTCCTCATC |
| 9 | *FOXA2* | NM_021784 | Forward: GGAACACCACTACGCCTTCAAC Reverse: AGTGCATCACCTGTTCGTAGGC |
| 10 | *DAT* | NM_001044 | Forward: CCTCAACGACACTTTTGGGACC Reverse: AGTAGAGCAGCACGATGACCAG |
